# Effect of surfactant types on particle size and morphology of flame-retardant zinc borate powder

**Published:** 2020-02-11

**Authors:** Yeliz İPEK

**Affiliations:** 1 Department of Chemistry and Chemical Proces Technologies, Vocational School of Tunceli, Munzur University, Tunceli Turkey; 2 Rare Earth Elements Application and Research Center, Munzur University, Tunceli Turkey

**Keywords:** Zinc borate, particle morphology, nanomaterial, surfactant, particle size

## Abstract

Zinc borate is a boron-containing chemical material that is used to increase the flame retardancy of polymeric materials, dyes, cables, fabrics, carpets, and the internal parts of automobiles and planes. Commercially used zinc borate, which has the formula of 2ZnO·3B _2_ O _3_ ·7H _2_ O, has a particle size between 10 and 20 μm. However, recent studies have shown that nanosized flame retardants have more superior flame retardancy and less negative effects on mechanical properties than microsized flame retardants. Nanosized flame retardants disperse more homogeneously and even low quantities are sufficient to provide high flame resistance. In this study, nano zinc borate powder was synthesized by a wet chemical method and the effects of nonionic, anionic, and cationic surfactants on the particle size and morphology of the zinc borate particles were investigated. Chemical purity and physical structures of the synthesized zinc borate powder were analyzed by XRD, FTIR, TG-DTA, TEM, and Zetasizer. The analysis results showed that the zinc borate powder had a chemical formula of 2ZnO·3B _2_ O _3_ ·7H _2_ O. TEM and Zetasizer results indicated that the nano zinc borate powder, which had nanoscale particle size distribution with needle- and flake-like structures, was synthesized using nonionic, anionic, and cationic surfactants.

## 1. Introduction

Nanosized flame retardants have significant importance for developing flame-retardant materials, especially for applications requiring high fire resistance and high mechanical properties. However, flame retardants with nanodimensions are important because they provide high performance at low amounts and do not cause a negative impact on the mechanical and chemical properties of the final product. Among a variety of flame retardants, zinc borate has attracted attention and a wide variety of studies have been conducted due to its properties such as smoke suppression during combustion, ineffectiveness on product appearance, nontoxic and noncarcinogenic qualities, and emission reduction [1,2].

The wet precipitation method is a low-cost and repeatable route. Using this method, the morphology and particle size of nanostructures can be controlled, especially by utilizing various precipitating agents and surfactants [3]. One of the most well-known methods for producing nanosized powder is to add surfactants to the solution medium. Thus, the surface of the particles formed is wrapped by the surfactant to prevent agglomeration. Zheng et al. synthesized nano zinc borate powder of about 1 μm in length and 50–100 nm in diameter to make a composite of polyethylene-nano zinc borate [1]. Ting et al. synthesized amorphous nano zinc borate powder with a rod-like shape and added polypropylene and high-density polyethylene [2]. They used ammonia, zinc nitrate, and borax as raw materials. Since the ammonia solution solved the zinc borate powder, vaporization of the ammonia led to precipitation of the zinc borate as nanometer-scaled powder. Tian et al. produced hydrophobic zinc borate using the chemical precipitation method [4]. Hydrophobic properties of the powders were provided with the addition of oleic acid to media during the process. Köytepe et al. used the microemulsion method for the synthesis of nano zinc borate powder, and to control the size of the substances, they added nonionic surfactant Span80 to the emulsion [5]. Ting et al. solved the precursor materials via the addition of an ammonia solution to create a homogeneous medium, then precipitated nano zinc borate powder by slowly removing the ammonia from the solution [6]. Dong et al. achieved 20- to 50- nm-sized nano zinc borate powder using the ethanol supercritical fluid drying method [7]. Baltaci et al. used cumene terminated poly(styrene-co-maleic anhydride) (PSMA) surfactant-modified zinc borate in PET to obtain polymer composites with improved flame retardancy and mechanical properties [8]. The composites obtained were characterized in terms of flame retardancy, mechanical properties, and morphology. Zinc borate particles obtained via the addition of PSMA (0.1% and 1%) had a smaller size than unmodified zinc borate. Li et al. stated that a zinc borate nano/microstructure with different morphologies had successfully been fabricated through a facile surfactant assisted method [9]. Their product had an effect on polyethylene, especially when the zinc borate was modified by oleic acid. Tian et al. detected that the optimal amount of oleic acid used as a surfactant was 1.0% of the weight of the synthesized zinc borate [10]. However, the effect of different surfactant types on the particle size and morphology of flame-retardant zinc borate powder has not yet been reported in the literature.

In the present study, the effect of the addition of different types of surface-active agents (nonionic, anionic, and cationic surfactants) on the particle size and shape of zinc borate powder was investigated. Zinc borate powder, which has a chemical formula of 2ZnO·3B _2_ O _3_ ·7H _2_ O, was produced on a nano scale. The production process of nano zinc borate was performed at low cost using a simple chemical method in terms of compliance with industrial production. The powder was synthesized via the wet chemical precipitation method using an ammonia solution, surfactant, zinc nitrate hexahydrate (Zn(NO _3_ ) _2_ ·6H _2_ O), and borax decahydrate (Na _2_ B _4_ O _7_ ·10H _2_ O). The cationic surface-active agent CTAB, nonionic surface active agent Triton-114, and oleic acid, which is an anionic surface-active agent, were used to investigate the effect of different types of surfactants on the physical structure and particle size of the zinc borate powder.

## 2. Experimental

**Figure 1:**
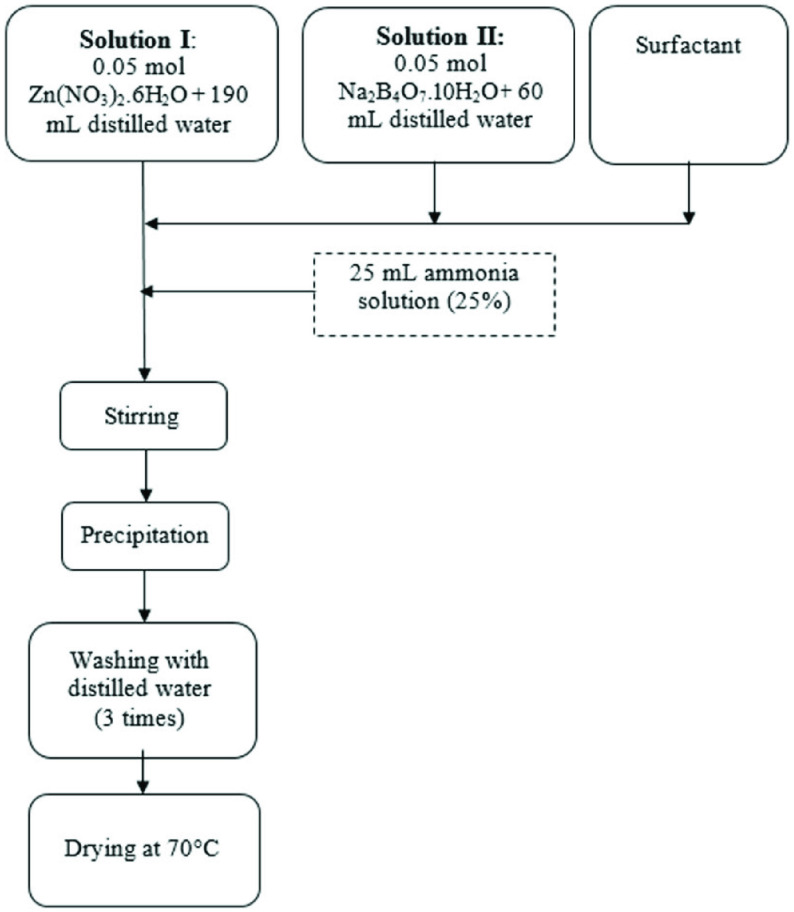
Process flow chart of the wet chemical method of zinc borate production.

The reagents used in this study were of analytical grade. Zinc nitrate (Zn(NO _3_ ) _2_ ·6H _2_ O, 98%; Merck, Darmstadt, Germany) and borax decahydrate (Na _2_ B _4_ O _7_ ·10H _2_ O, 99%; Eti Mine Works, Keçiören, Ankara, Turkey) were used as the zinc and boron sources. The Triton 114, oleic acid (≥99%), and cetyltrimethylammonium bromide (CTAB) were commercially available from Sigma-Aldrich (St. Louis, MO, USA) and used as the nonionic, anionic, and cationic surfactant materials, respectively. The samples were named CBA (without surfactant), CBA/CTAB (with CTAB), CBA/Triton-114 (with Triton-114), and CBA/Oleic acid (with oleic acid). To synthesize nanosized zinc borate powder, the wet chemical precipitation method was applied and the amount of surfactants used was 1.0% of the weight of the synthesized zinc borate. The process flow chart is given in Figure 1.

### 2.1. Synthesis

For the synthesis process, 2 precursor solutions were prepared. After dissolving zinc nitrate and borax decahydrate in distilled water separately, they were mixed and a white precipitate was formed instantaneously. Next, a certain amount of ammonia solution (25% concentrated ammonia, Merck) was added to the mixture to adjust the pH of the solution to 11. In this point, the precipitates were dissolved and a clear solution was obtained. The clear solution was mixed at 300 rpm in a batch-type glass reactor that had a heating jacket at 45 ◦ C for 5 h. Ammonia slowly evaporated, and when the pH of the mixture reached 10, zinc borate powder began precipitating as a fine powder. The reaction mechanism is as given below:

(1)Na2B4O5(OH)4.8H2O(s)→2Na+(aq)+B4O5(OH)42-(aq)+8H2O(l)(2)Zn(NO3)2.6H2O(l)(3)B4O5(OH)42-(aq)+Zn2+(aq)+H2O(l)→Zn[B4O5(OH)4].H2O(s)(4)Zn[B5O5(OH)4].H2O(s)+2H2O(l)→Zn[B3O3(OH)5].H2O(s)+B(OH)3(s)(5)Zn[B3O3(OH)5].H2O(s)→Zn[B3O3(OH)5](s)+H2O(l)

The resultant powder was washed 3 times with distilled water, and after filtration, the cake was dried in an oven at 70 ◦ C to obtain pure zinc borate powder.

### 2.2. Characterization

XRD measurements were performed using a Rigaku X-ray diffractometer (Tokyo, Japan) using Cu-Kα radiation at a scan rate of 1◦ /min between 5◦ and 60◦ (2θ) . In order to determine the percentage of weight loss and thermal behavior, differential thermal analysis and thermogravimetric analysis (TG-DTA) was performed using a NETZSCH STA 409C/CD (Selb, Germany) at a heating rate of 10 ◦ C/min, between 25 and 550 ◦ C, and under a N _2_ atmosphere. FTIR spectroscopy was performed to determine the O-H and B-OH interactions in the sample at a wave number range of 4000 to 650 cm ^-1^ using a Thermo Nicolet 6700 Model FTIR (Waltham, MA, USA). The particle size of the zinc borate powders and their morphologies were examined using transmission electron microscopy (TEM, 200 V; JEOL, Tokyo, Japan) and a Malvern Nano Zetasizer (Malvern, UK).

## 3. Results and discussion

**Figure 2:**
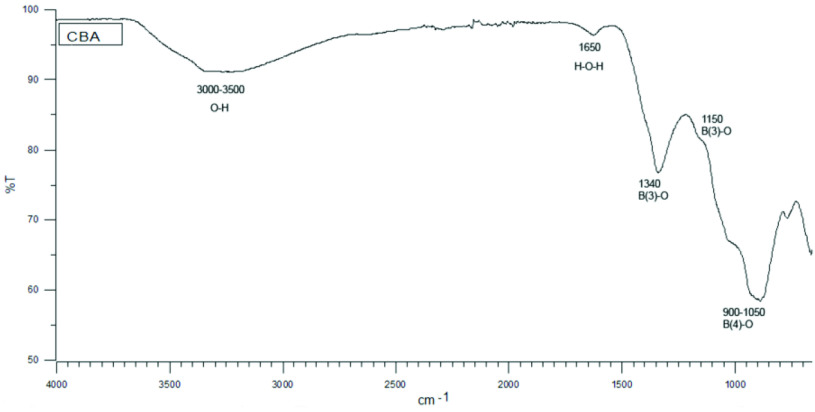
FTIR spectrum of CBA powder produced without surfactant.

The FTIR spectrum of the zinc borate powder (Figure 2), which was synthesized from zinc nitrate hexahydrate and borax decahydrate without surfactant at 45 ◦ C and 300 rpm for 5 h, exhibited an absorption band at about 3000–3500 cm ^-1^ , corresponding to the O–H group stretching vibration. The peak at 1650 cm ^-1^ pointed to formation of the H–O–H bending mode due to the crystal water included in the zinc borate. The peaks at 1340 cm ^-1^ , 1150 cm ^-1^ , and 675 cm ^-1^ were attributed to B(3)–O bonds and the peaks observed between 900 and 1050 cm ^-1^ were attributed to B(4)–O bonds [11–15].

**Figure 3:**
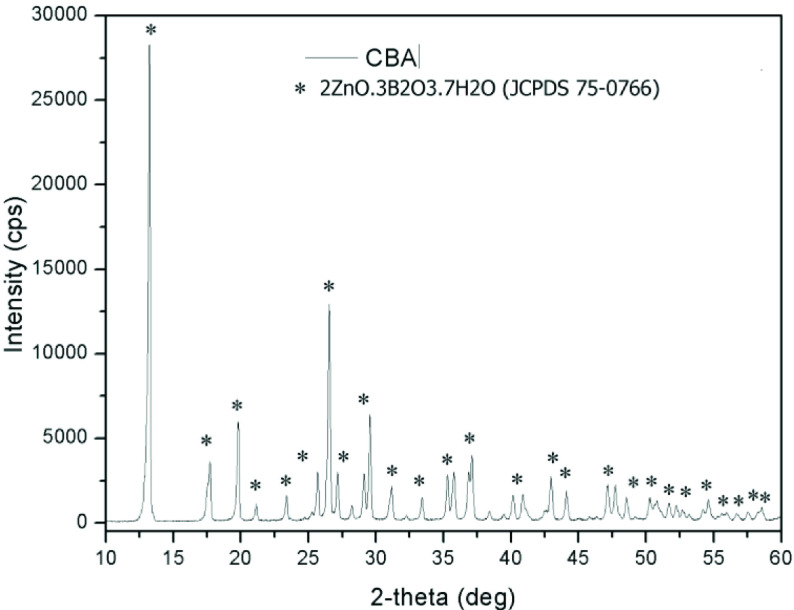
XRD pattern of CBA powder produced without surfactant.

The CBA powder crystal structure was investigated via XRD analysis. As shown in Figure 3, a crystalline phase was achieved. Major peaks at 13.2◦ , 17.5◦ , 19.8◦ , 21.2◦ , 23.4◦ , 26.5◦ , 28.5◦ , 30.9◦ , 33.4◦ , 35.3◦ , 37.4◦ , 40.2◦ , 41.1◦ , 43.3◦ , 44◦ , 47.2◦ , 50.3◦ , and 54.9◦ were observed. The crystal structure diffraction overlapped with the zinc borate, which had the formula 2ZnO.3B _2_ O _3_ ·7H _2_ O (JCPDS 75-0766). The crystal structure of the CBA powder exhibited pure zinc borate peaks [16,17]. The purity of the powder was achieved by washing the byproducts 3 times with distilled water after synthesis. The theoretical density was calculated as 4.6 g/cm ^3^ by proportioning the weights of the atoms in the crystal to the crystalline volume.

**Figure 4:**
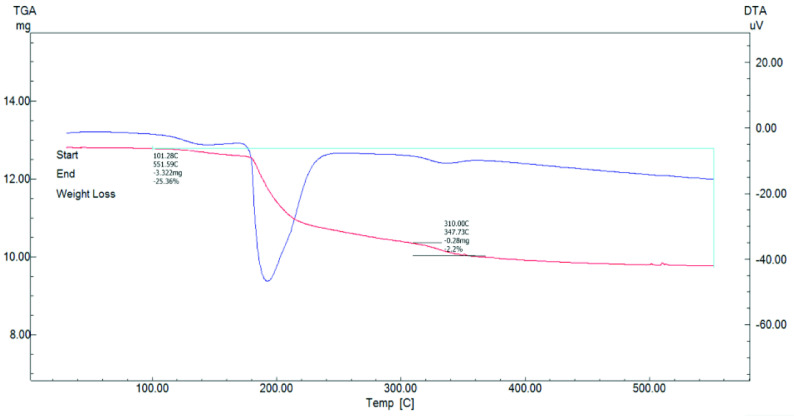
TG-DTA analysis of CBA powder produced without surfactant.

TG-DTA of the CBA powder indicated that the powder had 25.3% weight loss between 101 and 550 ◦ C (Figure 4). Zinc borate has structures with many different formulations, such as 4ZnO·B _2_ O _3_ ·H _2_ O, ZnO·B _2_ O _3_ ·1.12H _2_ O, ZnO·B _2_ O _3_ ·2H _2_ O, 6ZnO·5B _2_ O _3_ ·3H _2_ O, ZnO·5B _2_ O _3_ ·4.5H _2_ O, 2ZnO·3B _2_ O _3_ ·7H _2_ O, 2ZnO·3B _2_ O _3_ ·3H _2_ O, 2ZnO·3B _2_ O _3_ ·3.5H _2_ O, and 3ZnO·5B _2_ O3·14H _2_ O. The water content value was determined with TG-DTA in the literature. Gao and Liu [18] stated that the total weight loss between 138 and 700 ◦ C was 25.9% and this value corresponded to the water content of the molecule with the formula 2ZnO·3B _2_ O _3_ ·7H _2_ O. The theoretical crystal water weight of zinc borate, with the formula 2ZnO·3B _2_ O _3_ ·7H _2_ O, is 25.3% and the weight loss between 101 and 550 ◦ C was measured as 25.36%. Thus, the TG-DTA results exhibited good consistency with the thermal behavior properties of zinc borate powder, which has the chemical formula 2ZnO·3B _2_ O _3_ ·7H _2_ O [18]. The XRD, FTIR, and TG-DTA results supported each other according to the chemical structure and crystal water content. Weight losses occurred at several stages. Low weight loss up to 101 ◦ C was due to the evaporation of adsorbed water (free water). The endothermic peak for the main weight loss was seen at about 200 ◦ C (loosely bounded water) and between 310 and 350 ◦ C (tightly bounded water) it was related to the crystal water evaporation of the zinc borate powder [19–23].

**Figure 5:**
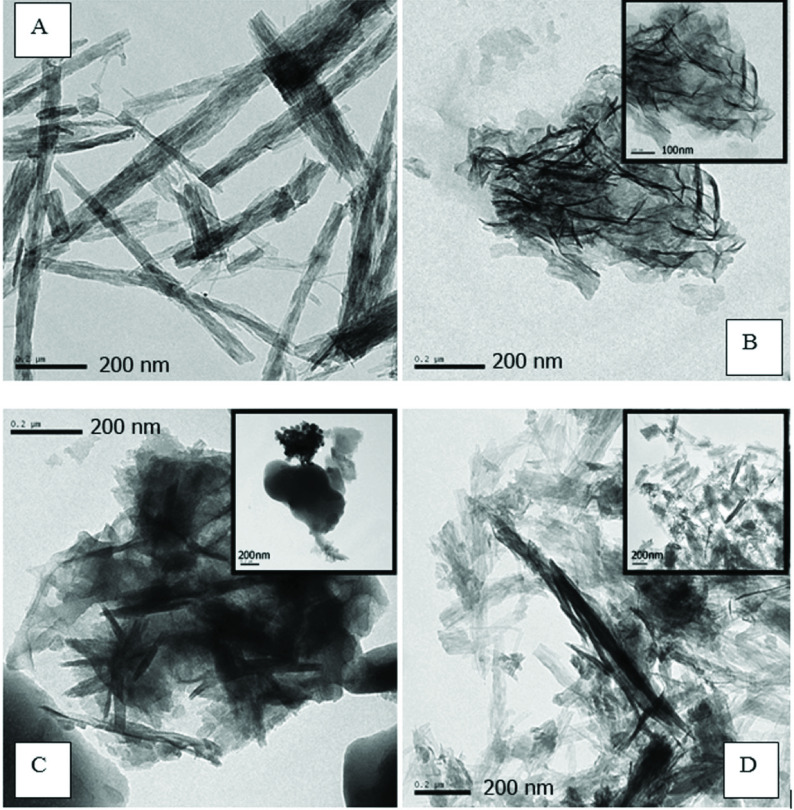
TEM images of nano zinc borate powders produced (a) without surfactant (CBA) and with the addition of (b) CTAB (CBA/CTAB), (c) Triton-114 (CBA/Triton-114), and (d) oleic acid (CBA/Oleic acid).

Zinc borate powder produced by the addition of nonionic, anionic, and cationic surfactants had identical XRD, FTIR, and TG-DTA results. To investigate the effect of different types of surfactants on the particle size and shape of zinc borate powder, TEM was used (Figure 5). TEM micrographs of the zinc borate powder that was produced without the addition of a surfactant revealed that rod-shaped particles or straps were formed (Figure 5a). While the thickness of the zinc borate rods was less than 50 nm, the length of the particles exceeded 1 μm. With the addition of cationic surface-active agent (CTAB), the straps were converted to needle-like particles with a length of about 50–200 nm and diameter of about 5–10 nm (Figure 5b). The addition of nonionic surface-active agent Triton-114 also resulted in needle-like particles with greater length and diameter than the powders produced with the addition of CTAB (Figure 5c). Particles that were 15–40 nm thick and 150–350 nm in length were obtained with the addition of Triton-114. The addition of oleic acid, which is an anionic surface-active agent, resulted in shorter straps of zinc borate particles when compared to the powder produced without the addition of a surfactant (Figure 5d). The thickness of the rods produced by the addition of oleic acid was less than 5–10 nm, while the length of the rods was less than 350 nm. Although the surfactants led to the formation of shorter and thinner rod-like particles, they did not prevent agglomeration of the particles. Thickness and length data of the synthesized powders are given in the Table.

**Table:** Thickness and length data of the synthesized powder measured with TEM and Zetasizer.

	Needle-like particles		Flake-like particles	Mean particle size of the synthesized powder measured with Zetasizer (nm)
Sample	Thickness, nm	Length, nm	Diameter, nm	
CBA	< 50	>1000	-	211
CBA/CTAB	5-10	50-200	25	66
CBA/Triton-114	15-44	150-350	30	20
CBA/Oleic acid	5-10	350	-	17

**Figure 6:**
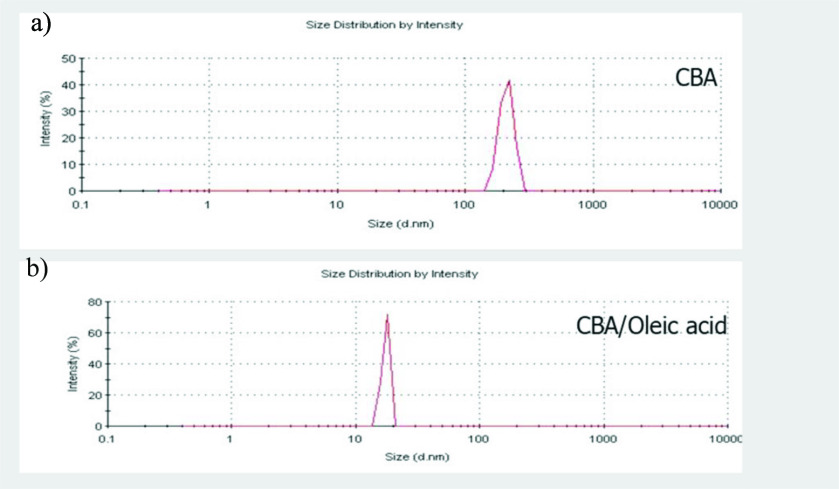
Particle size distribution of CBA and CBA/Oleic acid powder.

Zeta potential particle size measurements were applied to the samples to observe the particle size distribution with another method. Particle size distribution diagrams of the CBA and CBA/Oleic acid are given in Figure 6. The particle size of the CBA powder exhibited a homogeneous distribution (only 1 peak was observed) between 160 and 300 nm with a mean particle size of 211 nm, as shown in Figure 6a. The zinc borate powder synthesized with oleic acid as a surfactant also had a homogeneous particle size distribution between 11 and 20 nm (Figure 6b). The mean particle size of the CBA/Oleic acid powder was 17 nm. The mean particle sizes of the zinc borate particles produced with and without the addition of a surfactant are compared in the Table.

These results indicated that the TEM analysis and zeta potential particle size measurements were not very compatible. TEM analysis showed that the particle shape of the CBA was needle-like. Nanosized zinc borate powder, which was synthesized with different types of surfactants, formed as shorter and thinner rods or flake-like particles.

In this study, nanosized zinc borate powder was produced using the wet chemical precipitation method via the evaporation of ammonia with a surfactant present in the reaction medium. XRD and FTIR results showed that zinc borate was successfully synthesized. Moreover, the crystal water content of the powder was investigated via TG-DTA, which was consistent with zinc borate powder that had the chemical formula 2ZnO·3B _2_ O _3_ ·7H _2_ O. Dehydration temperature is an important parameter if the synthesized samples are proposed to be used as flame-retardant additives for polymers. The zinc borate synthesized in this study had a dehydration temperature of approximately 180–210 ◦ C, which was higher than the processing temperature of most polymers. Some polymers with processing temperatures lower than 180 ◦ C include poly(1-butene) [24], poly(ethylene adipate) [25], poly(ethylene oxide) [26], poly(methyl methacrylate) [27], and poly(propylene) [28]. The TEM investigation showed that surfactant-free synthesis resulted in strap widths of less than 50 nm and lengths more than 1 μm. The addition of oleic acid decreased the length and width of the rod-like particles. However, the addition of CTAB and Triton-114 resulted in a mixture of needle- and flake-like particle formation. While microsized particles were obtained with the conventional wet precipitation method, nanoparticles were available with the use of surfactants. Zetasizer analysis exhibited the lowest particle size, at 17 nm, with the addition of oleic acid as a surfactant during the synthesis of zinc borate. This result was obviously different from the TEM particle size result. Zetasizer analysis was applied for particles in aqueous media. This was attributed to the shape of the particles. The particles were generally needle- or rod-like and the position of the particle in the solution against the light source of the device affected the measurement results. Measuring spherical particles with a Zetasizer will give more accurate results. The reason for the different measurement results for the same sample lies behind the measurement principles of the instrument. The particle size obtained with the Zetasizer was the volume-weighted mean diameter, and it was assumed that the particles were spherical. However, TEM takes photos of the particles using an electron beam that has a very short wavelength emitted from a tungsten filament, which is transmitted through the sample. Since the particles were rod- or flake-like, TEM seemed to be more reliable to investigate the particle size and shape of the zinc borate samples.

In summary, in this study, it was shown that the addition of a surfactant had a clear effect on the zinc borate particle size and powder morphology. Oleic acid, a nonionic surfactant, was more effective than the other types of surfactants for reducing particle size and forming a uniform morphological structure.
